# Supplementary material for: Construction of management tools for early warning of prevacuum steam sterilization failure
Source: PLoS One. 2025 Jan 6;20(1):e0316322. doi: 10.1371/journal.pone.0316322 (PMC11703070; doi:10.1371/journal.pone.0316322)
Supplement: Attachment — Submitted filename: PONE.pdf [file pone.0316322.s003.pdf]

REVIEWER'S OPINION - PONE-D-24-20423

GENERAL CONSIDERATIONS

Originality: High
Thematic Depth: High
Thematic relevance: High
Article construction: Insufficient
Article composition: Incomplete
Writing rules: Appropriate

Conclusion:
(_) Rejected
(_) Accepted with minor adjustments
(x) Accepted with major adjustments
(_) Approved

Descrição dos itens:

**Item**	**Assessment**	**Observations**
Title	Confused	I suggest replacing “Construction and practice [...]” with “Construction and clinical validation [...]”. The purpose of the substitution is to clarify the study method employed.
Abstract	Incomplete	The objective is incomplete; it is evident that a mixed-methods study was employed, apparently a methodological study followed by a content validation and clinical validation. Clarify this. The employed method also lacks further explanations, even if they seem obvious. What is the study design? How many and what were the phases of development? Based on what methodological framework? What do you consider “specialized consultation”? The results indicate six alert indicators in four categories; this is incomplete, write them out in full, both the indicators and the categories.
Keywords	Insufficient.	Insert two more descriptors with greater potential for generalization on the subject..
Introdution	Incomplete	Insert the state of the art on the subject, the justification, and the relevance of the study. The inserted objective deviates from what is mentioned in the summary and needs standardization, even if it seems obvious to you.
Method	Incoherent	The study design does not seem clear within the various types of studies that exist and needs to be revised. The methodological reference model used is not mentioned, and it is necessary to follow one for scientific standardization and methodological rigor. The number of phases is implied; make it evident. The recruitment process for specialists and their function is not clear. A data search for identifying the indicators was mentioned; however, the process mentioned earlier was the interview with specialists. Which is the correct method? The terms “control group” and “experimental group” are commonly used for experimental studies, and are not suitable for this study, evaluate the possibility of replacing it with a similar one.
Results	Incoherent.	Nine indicators appeared, differing from what is stated in the abstract; minor adjustment needed.
Discussion	Adequate	Objective and complete.
Conclusion	Adequate	Create a pamphlet regarding the conclusions of your study.
References	Adequate	All elements are consistent.

Other observations: The document has great social and scientific relevance; however, it presents inconsistencies, primarily in the method. I advise adhering to all pointed observations to achieve success in the approval process.
