# Supplementary material for: Construction of management tools for early warning of prevacuum steam sterilization failure
Source: PLoS One. 2025 Jan 6;20(1):e0316322. doi: 10.1371/journal.pone.0316322 (PMC11703070; doi:10.1371/journal.pone.0316322)
Supplement: Attachment — Submitted filename: Revision letter 20241115.docx [file pone.0316322.s004.docx]

Dear editor,

Thank you for correcting the article "Construction of management tools for early warning of prevacuum steam sterilization failure". We made targeted changes and enhancements to the article after carefully reviewing expert revision opinions, and we have noted the precise changes in the article for your evaluation. Now, the revision ideas and issues raised by the review specialists are described one by one.

**Reviewer 1:**

Comment 1: The topic of the article is very relevant, the study presented is quite detailed and the techniques are compatible with the intended results, but the authors should not only review the grammar, but also some typing errors throughout the text.

Response 1: Thank you for you comment, we had sent the manuscript to a professional paper polishing agencies, to check grammatical errors and optimize the quality of language expression

Comment 2:In addition, there are some errors throughout the text, for example: In the Abstract the author mention that they divided the indicators into 4 categories, but they only describe 3.

Response 2: Thank you for your comment, we had revised this mistake and checked the consistency of the preceding text.

Comment 3: On page 10, penultimate line: the indicator "very familiar" was repeated 3 times and one indicator was missing.

Response 3: Thank you for your comment, we had revised this mistake.

Comment 4: If the formula described is correct (Ca=(Ci+Cs)/2), then the result on the penultimate line of page 11 is not correct, despite being higher than required.

Response 4: Thank you for your comment, since the questionnaire for expert had been revised in this study, we confounded the results before and after adjustment. We had revised this mistake, and all the statistical results of the questionnaire were uniformly taken as three decimal places.

**Reviewer 2:**

Comment 1: I suggest replacing “Construction and practice [...]” with “Construction and clinical validation [...]”. The purpose of the substitution is to clarify the study method employed.

Response 1: We had revised the paper title.

Comment 2: The objective is incomplete; it is evident that a mixed-methods study was employed, apparently a methodological study followed by a content validation and clinical validation. Clarify this. The employed method also lacks further explanations, even if they seem obvious. What is the study design? How many and what were the phases of development? Based on what methodological framework? What do you consider “specialized consultation”? The results indicate six alert indicators in four categories; this is incomplete, write them out in full, both the indicators and the categories.

Response 2:We have revised the Objectives and methods section of the abstract to specify the objectives and methods of the study. The main objectives of the study, including the management tools for the expected construction and the controlled trials, are introduced in the objectives, and the phases of the study and the content planned to be completed in each phase are introduced in the Methods of the abstract. The presentation of indicators and categories was also corrected

Comment 3: Insert two more descriptors with greater potential for generalization on the subject.

Response 3: We have added three keywords, namely Management tool; Safety margin; Expert consultation

Comment 4: Insert the state of the art on the subject, the justification, and the relevance of the study. The inserted objective deviates from what is mentioned in the summary and needs standardization, even if it seems obvious to you.

Response 4: We systematically adjusted the background section, adding new content and references around the recent progress of the research topic and the need for research,

Comment 5: The study design does not seem clear within the various types of studies that exist and needs to be revised. The methodological reference model used is not mentioned, and it is necessary to follow one for scientific standardization and methodological rigor. The number of phases is implied; make it evident. The recruitment process for specialists and their function is not clear. A data search for identifying the indicators was mentioned; however, the process mentioned earlier was the interview with specialists. Which is the correct method? The terms “control group” and “experimental group” are commonly used for experimental studies, and are not suitable for this study, evaluate the possibility of replacing it with a similar one.

Response 5:In this study, expert consultation method was used as the main method to reach a multidisciplinary consensus on the failure risk checklist and the calculation model of early sterilization failure risk. The failure risk checklist was selected and included inspection items unanimously approved by experts based on the results of previous studies. The calculation model of early sterilization failure risk was based on the margin of safety theory in industrial manufacturing, and a consensus was reached on the calculation formula for early warning of sterilization failure using the temperature and pressure sensor readings of pressure sterilizers. In addition, we changed the names of "control group" and "experimental group" to "test group" and "conventional group" during the method validation process.

Comment 6: Nine indicators appeared, differing from what is stated in the abstract; minor adjustment needed.

Response 6: Thank you for pointing out the error in this article, we have corrected it

Response 7: Create a pamphlet regarding the conclusions of your study.

Response 7: We systematically adjusted the conclusions and added summary statements
